# Transcriptomic and metabolomic data of *Populus deltoides* ‘Shalinyang’ response to feeding of *Anoplophora glabripennis* adults

**DOI:** 10.3389/fpls.2026.1782656

**Published:** 2026-03-13

**Authors:** Hui-Quan Sun, Yu-Rong Ren, Ying Yang, Zhi Su, Li-Jun Dong, Jian-Feng Liu, Jian-Rong Wei

**Affiliations:** 1 School of Life Sciences/Hebei Basic Science Center for Biotic Interaction, Hebei University, Baoding, Hebei Province, China; 2 Engineering Research Center of Ecological Safety and Conservation in Beijing-Tianjin-Hebei (Xiong’an New Area) of MOE, Baoding, Hebei Province, China; 3 Experimental Center of Desert Forestry, Chinese Academy of Forestry, Dengkou, Inner Mongolia, China

**Keywords:** *Anoplophora glabripennis*, metabolomic, multi-omics analysis, *Populus deltoides* ‘Shalinyang’, transcriptomic

## 1 Introduction

Poplar trees have been selected as the primary species for constructing protective forests due to their comprehensive advantages in crown width, height, growth rate, drought resistance, and cold tolerance compared to other tree species in the northwestern region of China (Luo et al., 2002). However, establishing these protective forests has been severely impacted by the infestation of *Anoplophora glabripennis* Motschulsky (Coleoptera: Cerambycidae). This destructive wood-boring beetle, native to China and the Korean Peninsula (Williams et al., 2004; Straw et al., 2016), is also called the Asian longhorned beetle (ALB). Since the late 20th century, it has expanded its range to North America and Europe (Hérard et al., 2009) and is widely acknowledged as a significant pest of international quarantine concern (Allison et al., 2004; Hu et al., 2009; Haack et al., 2010; Meng et al., 2015; Xu and Teale, 2021; Zhou et al., 2021). Despite extensive research on controlling ALB, its broad range of host trees and cryptic behavior make it particularly difficult to manage (Haack et al., 2010). In this context, *Populus deltoides* ‘Shalinyang’ (PdS), a newly developed stress-resistant poplar cultivar developed by the Chinese Academy of Forestry for northwestern China, has shown good biological traits and has demonstrated a strong inhibitory effect on ALB larval growth and adult lifespan (Cao et al., 2016; Wang et al., 2018; Sun et al., 2024, Sun et al., 2026). Therefore, how to use this excellent biological trait to control ALB is a question worthy of in-depth investigation.

Studies demonstrate that herbivore attack triggers dynamic adjustments in plants, including coordinated changes in transcriptomic and metabolic profiles. The development of transcriptomics and metabolomics also offers valuable opportunities to investigate the molecular mechanisms of plant resistance to herbivorous insect infestations (Ding et al., 2024; Li et al., 2024; Luo et al., 2025). Integrating these two omics technologies will also facilitate the identification of correlations between genes and metabolites during pest attacks on plants (Luo et al., 2025). To date, research on the insect resistance of PdS has focused on its interaction with ALB larvae. For example, Luo et al (Luo et al., 2024). attributed PdS’s insect resistance to the positive regulation of lignan biosynthesis by the *PdPLR1* gene in PdS after larval infection. He et al. (2025) and Wang et al. (2025a) also found that PdS’s insect resistance is associated with the positive regulation of jasmonic acid and salicylic acid biosynthesis. However, there is little information about how PdS defends against ALB adults at the molecular and biochemical levels.

To systematically compare differences between tissues under the same conditions and within the same tissue under different conditions, we utilized leaves and phloem of PdS before and after infested by ALB adults as our experimental materials. We employed non-targeted metabolomics methods to detect metabolites and utilized second-generation sequencing technology to obtain transcriptome sequencing datasets. A total of 12 samples produced 82.52 Gb of clean data and revealed 3,552 metabolites. These data enhance our understanding of the defense processes of PdS against the attacks by ALB adults and also provides an essential reference for future research on the resistance mechanisms of poplar to beetles and the breeding of new insect-resistant poplar varieties.

## 2 Materials and methods

### 2.1 Plants, insects, and plant treatments

The PdS used in the study was sourced from the Experimental Center of Desert Forestry of the Chinese Academy of Forestry. The source of *A. glabripennis* was provided by Ecology and Nature Conservation Institute of Chinese Academy of Forestry. The cultivation of these poplars and beetles, as well as the investigation, was conducted in the experimental greenhouse of the School of Life Sciences, Hebei University. Male and female adults starved for 24 hours were randomly assigned to poplars under similar growth conditions, with one male and one female beetle on each PdS. The no-insect treatment was used as the control group. After 48 hours of ALB feeding, leaves and phloem of PdS (at the boundary between ALB-feeding and non-feeding areas) were collected, and samples from the control group were collected from the same part. Since this study focuses on the overall metabolic phenotype caused by the actual damage to PdS from ALB feeding and the characteristics of ALB adult damage to PdS, which are difficult to accurately reproduce through artificial simulation, mechanical damage was not considered as another control group. The samples were frozen in liquid nitrogen and stored at -80°C for subsequent analysis. In this study, three biological replicates were performed in both the treatment and control groups to ensure the reliability of the test results.

### 2.2 Physiological index measurement

The sample was ground into a fine powder using liquid nitrogen, and 0.1 g sample was weighed and placed in a centrifuge tube. The superoxide dismutase (SOD) activity assay kit, catalase (CAT) activity assay kit, peroxidase (POD) activity assay kit, and malondialdehyde (MDA) content assay kit (Beijing Solarbio Science & Technology Co., Ltd., Beijing, China) were used for detection. Four replicates were prepared for each sample, and the procedures and calculation formulas were performed according to the kit instructions. These data were analyzed using Graphpad Prism 10.5 and presented as mean ± standard error (SE). Differences between treatments were compared using the Student’s t-test, with significance levels of * p < 0.05, ** p < 0.01, and *** p < 0.001.

### 2.3 RNA extraction, library preparation, sequencing, and transcriptome data analysis

Total RNA of plant samples from each treatment was extracted using RNApure Fast Plant Kit (CW0598S, CWBIO, China) according to the instructions in three biological replicates. Then, total RNA concentration was determined using a NanoDrop spectrophotometer (ThermoFisher Scientific, USA), and the integrity of the purified RNA was assessed on a 1% agarose gel.

The RNA-seq transcriptome library was prepared following Illumina^®^ Stranded mRNA Prep, Ligation (San Diego, CA) using 1 μg of total RNA by Shanghai Majorbio Bio-pharm Bio-technology Co., Ltd. (Shanghai, China). After quantification by Qubit 4.0, the library was sequenced on the NovaSeq™ X Plus sequencing platform. The obtained raw data were filtered using fastq (Chen et al., 2018). After removing low-quality raw reads, the clean reads were mapped to the *Populus trichocarpa* v4.1 as a reference genome using HiSat2 (Kim et al., 2015).

We designated and categorized the treatment and control groups based on the feeding locations of ALB on PdS as follows: feeding on the phloem (TP) and feeding on the leaves (TL). The corresponding control groups were named CKP and CKL, respectively. Both transcriptomic and metabolomic analyses were conducted using this grouping framework. Gene abundances were quantified using RSEM (Li and Dewey, 2011), followed by differentially expressed analysis using DESeq2 (Love et al., 2014). The screening criteria for differentially expressed genes (DEGs) were set to meet |log_2_FC| ≥ 1 and false discovery rate (FDR) < 0.05. To further elucidate the biological functions of differentially expressed genes (DEGs), Goatools (Klopfenstein et al., 2018) and Python scipy packages (http://scipy.org/install/) were used to perform Gene Ontology (GO, http://geneontology.org/) terms and Kyoto Encyclopedia of Genes and Genomes (KEGG, http://www.kegg.jp/) pathway enrichment analysis on DEGs, respectively.

To validate the transcriptome data, we randomly selected nine differentially expressed genes for qPCR analysis. cDNA was synthesized using the HiFiScript All-in-One RT Master Mix for qPCR Kit (CW3371, CWBIO, China). qPCR was performed using the Tli RNaseH Plus kit (RR820A, Takara, Dalian, China) on a Roche LC96 Real-Time PCR System (Roche Diagnostics, Indianapolis, Indiana, USA). Thermal cycling conditions were as follows: initial denaturation at 95 °C for 2 min, followed by 45 cycles for 5 s at 95 °C, 58 °C for 30 s, and 72 °C for 30 s. Transcript levels of target genes were normalized to the geometric mean of reference genes and calculated using the 2^−ΔΔCT^ method. All reactions were performed with three biological replicates, and three technical replicates were performed for each replicate. All specific primers are listed in **Supplementary Table 1**.

### 2.4 Metabolite extraction and untargeted metabolomic data analyses

For each biological replicate, 100 mg of plant samples were placed in a centrifuge tube, added with 80% methanol buffer, and then placed in a frozen tissue grinder Wonbio-96c (Shanghai wanbo biotechnology co., LTD) for metabolite extraction. After low-temperature vortex centrifugation (13000 rpm, 15 min), the supernatant was collected as a sample. LC-MS/MS analysis of metabolites separated on an ACQUITY BEH C18 column (100 mm × 2.1 mm i.d., 1.7 µm; Waters, USA) was performed using the UHPLC-Q Exactive system. Then, the LC/MS raw data were converted into a three-dimensional data matrix in CSV format, including sample information, metabolite names, and mass spectral response intensities, using Progenesis QI (Waters Corporation, Milford, USA). Internal standard peaks and any known false-positive peaks (including noise, column bleed, and derivatized reagent peaks), were removed from the data matrix, redundant peaks were removed, and peak-pooling was performed. At the same time, MS and MS/MS mass spectrometry data were matched against the self-built plant-specific metabolite database (MJDBPM) of Majorbio Biotechnology Co., Ltd. (Shanghai, China) using Progenesis QI with an MS mass error threshold of <10 ppm and a specified list of common adduct ions. Metabolite annotations were also based on the fragment matching with available databases, especially Human Metabolome Database (HMDB) (https://hmdb.ca/) and KEGG (https://www.genome.jp/kegg), to maximize the number of metabolite identifications. Furthermore, for metabolite annotations, only features with MS2 information were included. metabolites were identified only when the fragmentation score relative to the self-built library was >35 or the theoretical fragmentation score matched against databases such as HMDB was >40. As part of the system conditioning and quality control process, three quality control (QC) samples were also prepared by mixing equal volumes of all samples, and their processing and testing methods were identical to those used for the analytical samples. Additionally, the internal standards exhibited a coefficient of variation (CV) of 2.52% to 11.96% across QC samples, further confirming the instrument’s stability (**Supplementary Table 2**).

Next, the data matrices obtained by searching the database from two columns were merged, deduplicated, and preprocessed. Specifically, at least 80% of the metabolic features detected in any set of samples were retained. Missing values were imputed with the minimum value in the data matrix, and the response intensities of the sample mass spectrometry peaks were normalized via summation normalization to minimize errors from sample preparation and instrument instability, yielding a normalized data matrix. Meanwhile, QC samples with relative standard deviation (RSD) > 30% were excluded, and the data were log10-transformed to obtain the final data matrix for subsequent analysis. Following the data matrix preprocessing, the principal component analysis (PCA) and orthogonal least squares discriminant analysis (OPLS-DA) were performed using the ropls package (Version 1.6.2) in the R language. Data were Pareto-scaled prior to modeling. The quality of the OPLS-DA models was assessed by the default seven-fold cross-validation, and the model performance parameters (R²X, R²Y, and Q²) were calculated. R²X and R²Y represent the fraction of the variance explained in the X and Y matrices, respectively, while Q² indicates the predictive ability of the model. To further guard against overfitting, a permutation test (n = 200) was performed, in which the class labels were randomly permuted 200 times, and the corresponding R² and Q² values of the permuted models were compared with those of the original model. Additionally, ANOVA (p-value) was employed to assess the statistical significance of the model. Differential expressed metabolites (DEMs) were identified using variable importance (VIP > 1) in the projection from the OPLS-DA model, along with the student’s t-test (p-value < 0.05). The identified DEMs were annotated with metabolic pathways using the KEGG pathway database (http://www.kegg.jp/kegg/pathway.html), and the DEMs were enriched for analysis using the Python package “scipy.stats” (http://docs.scipy.org/doc/scipy).

## 3 Preliminary data analysis

*P. deltoides* ‘Shalinyang’ (PdS), an important tree species for shelterbelts in the semi-arid and arid regions of northwestern China, has shown resistance to the destructive wood-boring beetle *A. glabripennis* (ALB). However, comprehensive research on PdS remains limited. This study compared PdS leaves and phloem tissues before and after ALB adult feeding, utilizing untargeted metabolomics (LC-MS/MS) and transcriptomics (RNA-Seq) methods.

**Figure 1:**
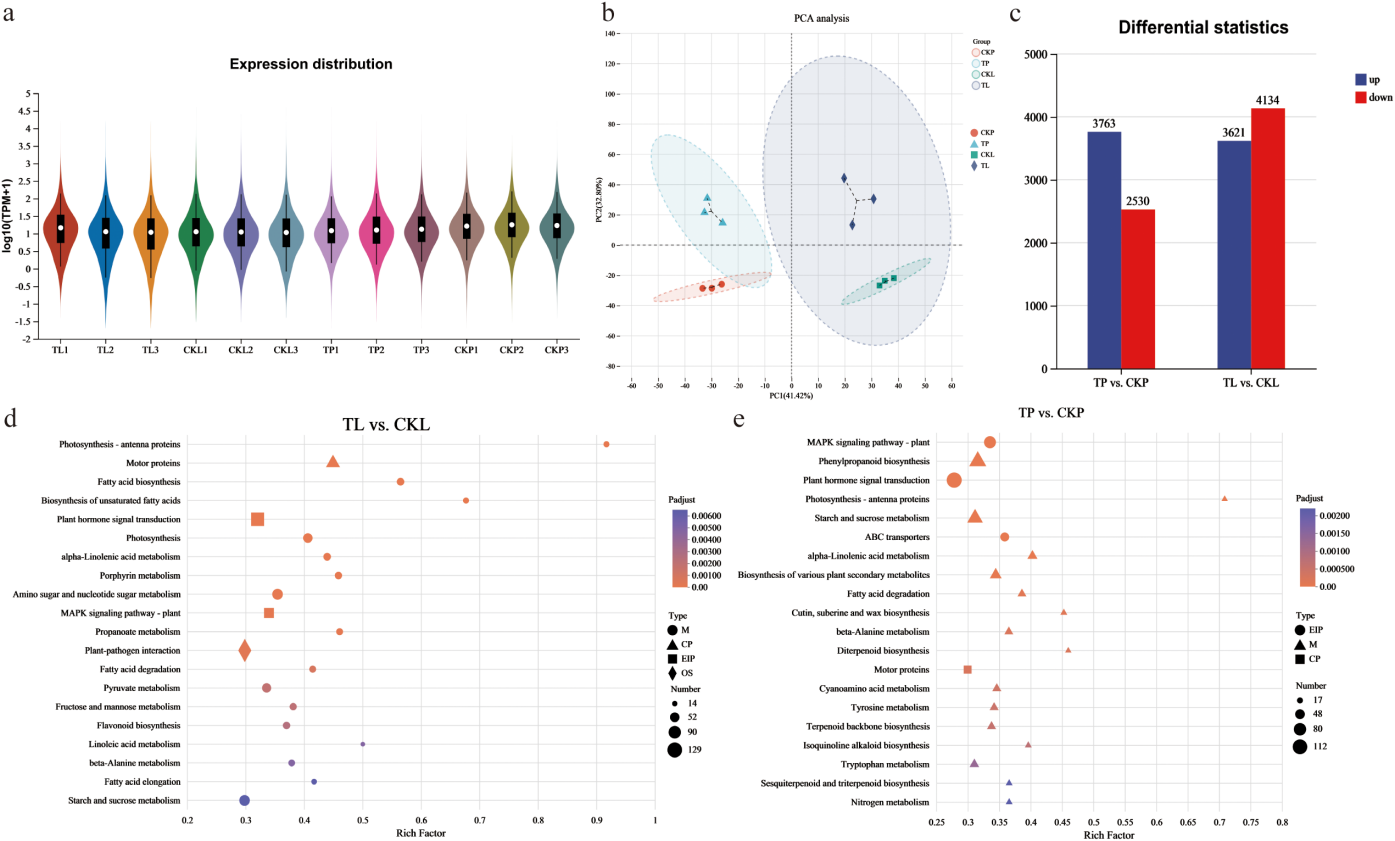
Quality control of transcriptome data and differentially expressed genes (DEGs) analysis for 12 *Populus deltoides* ‘Shalinyang’ samples. **(a)** Violin plot showing the distribution of gene expression. **(b)** PCA score plot illustrating expression profiles from various samples. **(c)** Overview of the DEGs among different treatment groups. **(d)** Statistical analysis of the enrichment of the top 20 KEGG pathways of the DEGs between TL vs. CKL. The x-axis represents the level of Rich factor, and y-axis represents the type of pathway. Dot size represents the number of DEGs, and colors indicate corrected p-values. The shape of the dot rep-resents the KEGG classification, specifically as follows: M: Metabolism, EIP: Environmental Information Processing, CP: Cellular Processes, OS: Organismal Systems. The same applies to graphs e. **(e)** Statistical analysis of the enrichment of the top 20 KEGG pathways of the DEGs between TP vs. CKP.

A total of 561,491,948 raw reads were obtained from the twelve samples in this study. Following filtering and error correction, 528,788,154 high-quality reads were retained for downstream bioinformatics analysis, yielding an average of 6.88 Gb of data per sample. The average quality scores, Q20 and Q30, were 98.89% and 96.29%, respectively, and the average GC content was 43.89%, indicating the high sequencing quality of this study (**Supplementary Table 3**). After quality control, the sequencing data were aligned to the reference genome. Of these, 78.65% (TP2) to 86.72% (CKL2) of the sequencing data aligned to the reference genome (**Supplementary Table 3**). By comparing with databases, a total of 34,699 genes with gene annotation information were discovered in this study (**Supplementary Table 4**). As illustrated in **Figure 1a**, the violin plot demonstrated the even distribution of expressed genes across each sample. Furthermore, the PCA results indicated that all arrays utilizing TPM values are presented in **Figure 1b**. The first two principal components accounted for 41.42% and 32.80%, respectively, demonstrating excellent separation effects. Additionally, two comparison groups (TP vs. CKP and TL vs. CKL) were selected for analysis. Among these, TP vs. CKP identified 6,293 DEGs, consisting of 3,763 upregulated and 2,530 downregulated DEGs. TL vs. CKL revealed 7,755 DEGs, with 3,621 upregulated and 4,134 downregulated DEGs (**Figure 1c**). Subsequently, KEGG enrichment analysis was performed on the DEGs in the two comparison groups. The results showed that DEGs in TP vs. CKP and TL vs. CKL were significantly enriched with 29 and 38 KEGG pathways, respectively (**Figures 1d, e**; **Supplementary Table 5**). To understand the biological functions of DEGs, we also analyzed all DEGs in the two comparison groups using the GO annotation system. The results showed that all DEGs of the two comparison groups were divided into 38 level-2 functional classification terms including 18 biological processes, 2 cellular components, and 18 molecular functions (**Supplementary Figure 1**; **Supplementary Table 6**). In addition, to verify the accuracy of the RNA-seq data, we analyzed the expression of nine genes using RT-qPCR. The results showed a similar trend (**Supplementary Figure 2**), further confirming the accuracy of the RNA-seq data.

**Figure 2:**
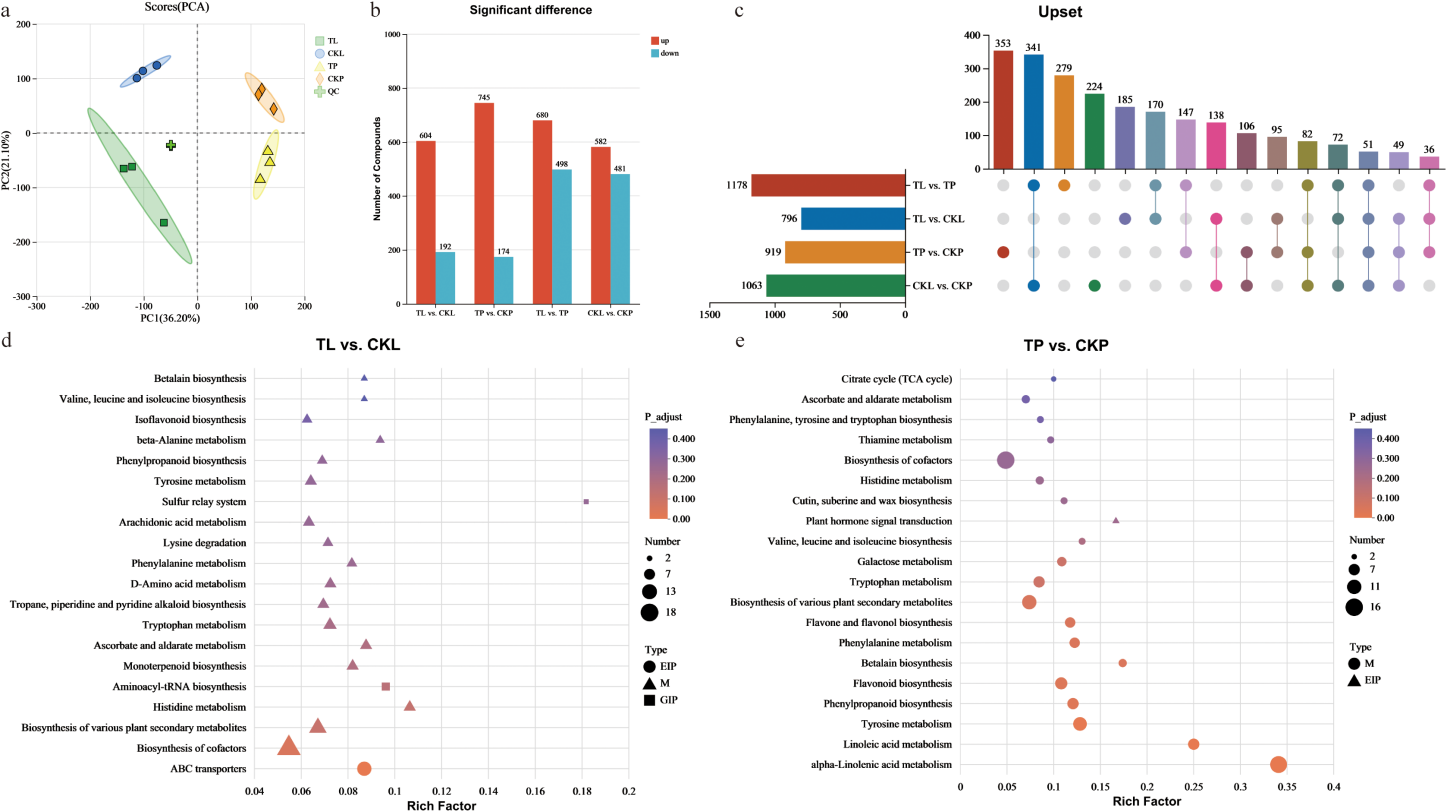
Quality control of metabolome data and differentially expressed metabolites (DEMs) analysis for 12 P*. deltoides* ‘Shalinyang’ samples. **(a)** PCA score plot showcasing expression profiles from various samples. **(b)** Statistical analysis of DEMs across different comparison groups. **(c)** Upset analysis of DEMs across different comparison groups. **(d)** Statistical analysis of the enrichment of the top 20 KEGG pathways of the DEMs between TL vs. CKL. The x-axis represents the level of Rich factor, and y-axis represents the type of pathway. Dot size represents the number of DEGs, and colors indicate corrected p-values. The shape of the dot rep-resents the KEGG classification, specifically as follows: M: Metabolism, EIP: Environmental Information Processing, GIP: Genetic Information Processing. The same applies to graphs e. **(e)** Statistical analysis of the enrichment of the top 20 KEGG pathways of the DEMs between TP vs. CKP.

In this study, samples for metabolome and transcriptome analysis were drawn from the same batch to ensure the reliability of the measurement results, yielding a total of 3,552 annotated metabolites (**Supplementary Table 7**). Differences in the metabolome between leaves and phloem before and after feeding were assessed using principal component analysis (PCA). The first two principal components, PC1 and PC2, accounted for 36.20% and 21.10% of the total variation, respectively (**Figure 2a**). Samples from different groups were separated, indicating significant metabolic differences. Additionally, we selected the same two control groups used in the transcriptomic analysis (TP vs. CKP and TL vs. CKL) for further examination, and an OPLS-DA model was constructed. The model’s parameter estimates, permutation test results, and ANOVA results confirmed its reliability in identifying discriminant metabolites (**Supplementary Figure 3**; **Supplementary Table 8**). Among these, 796 DEMs were identified in TL vs. CKL, and 919 DEMs in TP vs. CKP (**Figures 2b, c**). The results of the KEGG enrichment analysis indicated vigorous metabolic activity in both the leaves and phloem of PdS after feeding by ALB (**Figures 2d, e**; **Supplementary Table 9**). Simultaneously, the increased activities of POD, SOD, and CAT, as well as the increased MDA content in PdS after ALB ingestion in this study, also indicate PdS’s response to ALB (**Supplementary Figure 4**).

Furthermore, we integrated transcriptomic and metabolomic analysis in this study. The results showed that the ABC transporters pathway was the only pathway in which both DEGs and DEMs were significantly enriched after ALB consumed PdS leaves (**Supplementary Figure 5a**). And Phenylpropanoid biosynthesis, alpha-Linolenic acid metabolism, Biosynthesis of various plant secondary metabolites, and Tyrosine metabolism were pathways in which DEGs and DEMs were significantly enriched after ALB consumed PdS phloem (**Supplementary Figure 5b**). To further elucidate the regulatory relationships suggested by the KEGG enrichment analysis, we conducted pearson correlation analysis on the DEGs and DEMs in these 5 significantly enriched pathways, and the resulting correlation matrices were presented in heatmap form in **Supplementary Figure 6**. Among them, jasmonic acid content increased after beetles consumed the phloem, and the genes involved in jasmonic acid synthesis, including three lipoxygenase (*LOX2S*), one hydroperoxide lyase (*HPL*), three hydroperoxide dehydratase (*AOS*), two allene oxide cyclase (*AOC*), eight 12-oxophytodienoic acid reductase (*OPR*), two OPC-8:0 CoA ligase 1 (*OPCL1*), five acyl-CoA oxidase (*ACOX*), and one acetyl-CoA acyltransferase 1 (*ACAA1*), were all significantly upregulated. The **Supplementary Figure 6a** heatmap analysis revealed strong positive correlations between the expression of three *ACOX* genes (Potri.006G101800.v4.1, Potri.016G118000.v4.1, and Potri.013G123500.v4.1) and one *ACAA1* gene (Potri.001G051900.v4.1) and JA-Ile accumulation, providing strong evidence for coordinated regulation of this pathway at both the transcriptional and metabolic levels. These findings provide a more detailed view of potential regulatory points in the jasmonate biosynthesis pathway under our experimental conditions.

Overall, this study integrated transcriptomics and metabolomics analyses to examine the effects of ALB feeding on the leaves and phloem of PdS. These findings aid our understanding of the metabolic basis for PdS leaves and phloem defense mechanisms against ALB infestation. Furthermore, in recent years, there has been an increasing number of studies on the interaction between poplar trees and pests using transcriptomics and metabolomics (Ding et al., 2024; Jiang et al., 2024; Li et al., 2024; Pastierovič et al., 2024), but research on poplar tree resistance to the ALB remains relatively limited (Luo et al., 2024; Fu et al., 2025; Wang et al., 2025b).These data provide fundamental and valuable information for understanding how the PdS responds to ALB adult infestation. The relatively abundant transcriptome data provides a strong foundation for exploring metabolite formation, tissue-specific expression, and related studies. They can be utilized in future studies to explore the interaction mechanisms between poplar species and wood borers and investigate the insect resistance mechanisms of PdS.

## Data Availability

The current study deposited the raw metabolomics datasets in the Metabolights repository under the designated identifier MTBLS12296. The transcriptomic data can be retrieved from National Center for Biotechnology Information (NCBI) Sequence Read Archive (SRA) under the BioProject number PRJNA1232629 with accessions SRR32619380-SRR32619391.

## Supplementary material

Supplementary Figure 1 Statistical analysis of Gene Ontology (GO) classification of all differentially expressed genes (DEGs) before and after *Populus deltoides* ‘Shalinyang’ ingestion by *Anoplophora glabripennis.*

Supplementary Figure 2 Quantitative RT-PCR (qRT-PCR) validation of nine differentially expressed genes chosen from the transcriptome.

Supplementary Figure 3 OPLS-DA score and validation plots of the OPLS-DA model by permutation testing (n = 200) for pairwise comparisons among different groups.

Supplementary Figure 4 SOD activity (a), CAT activity (b), POD activity(c), and MDA(d) content of *P. deltoides* ‘Shalinyang’ leaves and phloem before and after ingestion by *A. glabripennis*.

Supplementary Figure 5 Co-enrichment pathways of DEGs and DEMs of *P. deltoides* ‘Shalinyang’ leaves(a) and phloem(b) before and after ingestion by *A. glabripennis*.

Supplementary Figure 6 Correlation heatmap between DEGs and DEMs across five significant co-enrichment pathways of *P. deltoides* ‘Shalinyang’ leaves and phloem before and after ingestion by *A. glabripennis*.

Supplementary Table 1 The primer sequence information for qRT-PCR.

Supplementary Table 2 Coefficients of variation for internal standards in quality control (QC) samples.

Supplementary Table 3 Transcriptomics data and mapping statistics of *P. deltoides* ‘Shalinyang’.

Supplementary Table 4 TPM values of all differentially expressed genes.

Supplementary Table 5 KEGG pathways were significantly enriched among differentially expressed genes in the two comparison groups.

Supplementary Table 6 level-2 GO functional classifications of differentially expressed genes in the two comparison groups.

Supplementary Table 7 Information on all annotated metabolites in the 12 samples.

Supplementary Table 8 Summary of OPLS-DA model parameters for pairwise comparisons among different groups.

Supplementary Table 9 KEGG significantly enriched pathways of differentially expressed metabolites in the two comparison groups.
