# Supplementary material for: Transcriptomic and metabolomic data of Populus deltoides ‘Shalinyang’ response to feeding of Anoplophora glabripennis adults
Source: Front Plant Sci. 2026 Mar 13;17:1782656. doi: 10.3389/fpls.2026.1782656 (PMC13023405; doi:10.3389/fpls.2026.1782656)
Supplement: Supplementary Figure 1 — Statistical analysis of Gene Ontology (GO) classification of all differentially expressed genes (DEGs) before and after Populus deltoides ‘Shalinyang’ ingestion by Anoplophora glabripennis. [file DataSheet1.docx]

Supplementary Material

## Supplementary Figures


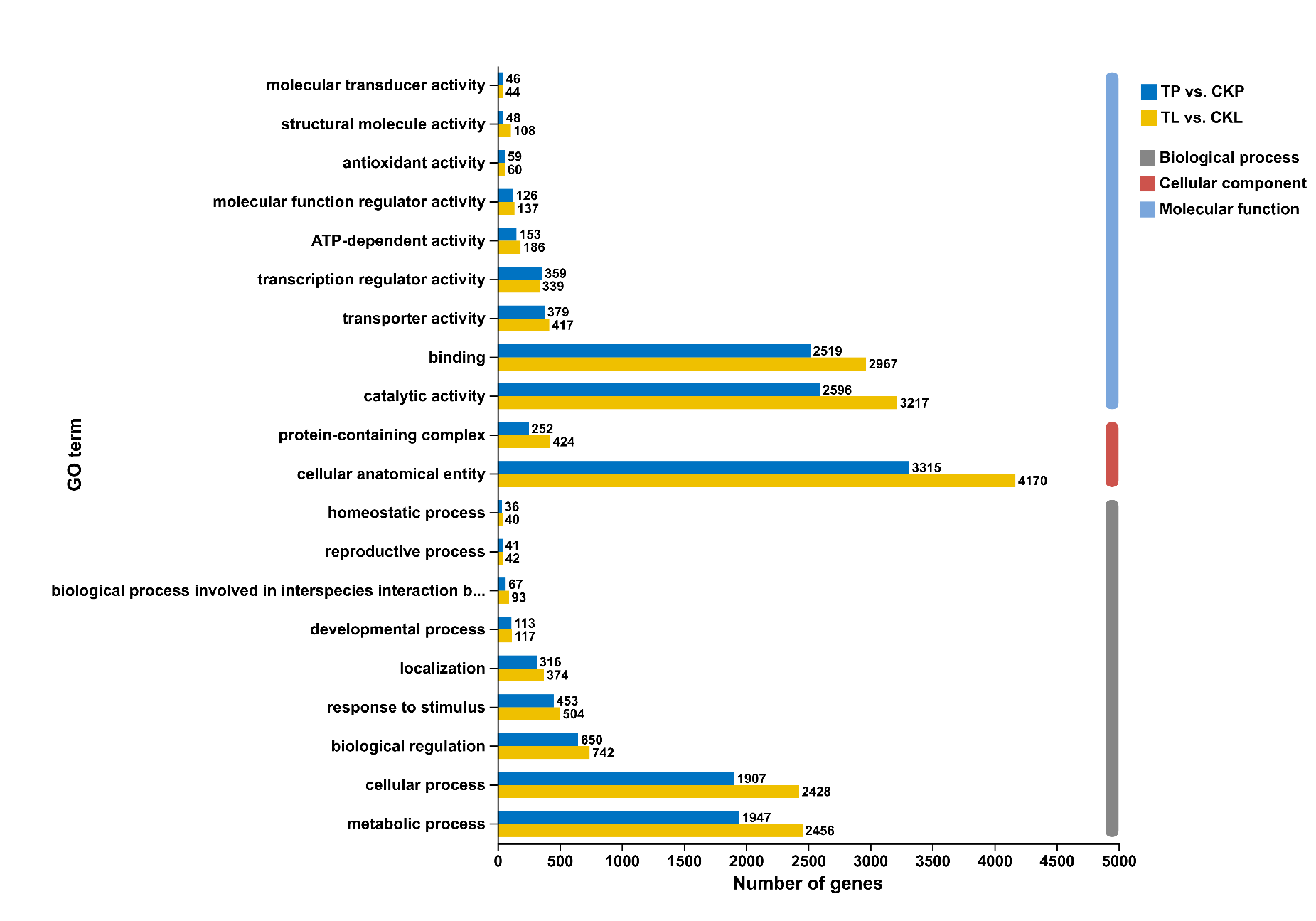


**Supplementary Figure 1.** Statistical analysis of Gene Ontology (GO) classification of all DEGs before and after *Populus deltoides* ‘Shalinyang’ ingestion by *Anoplophora glabripennis.*


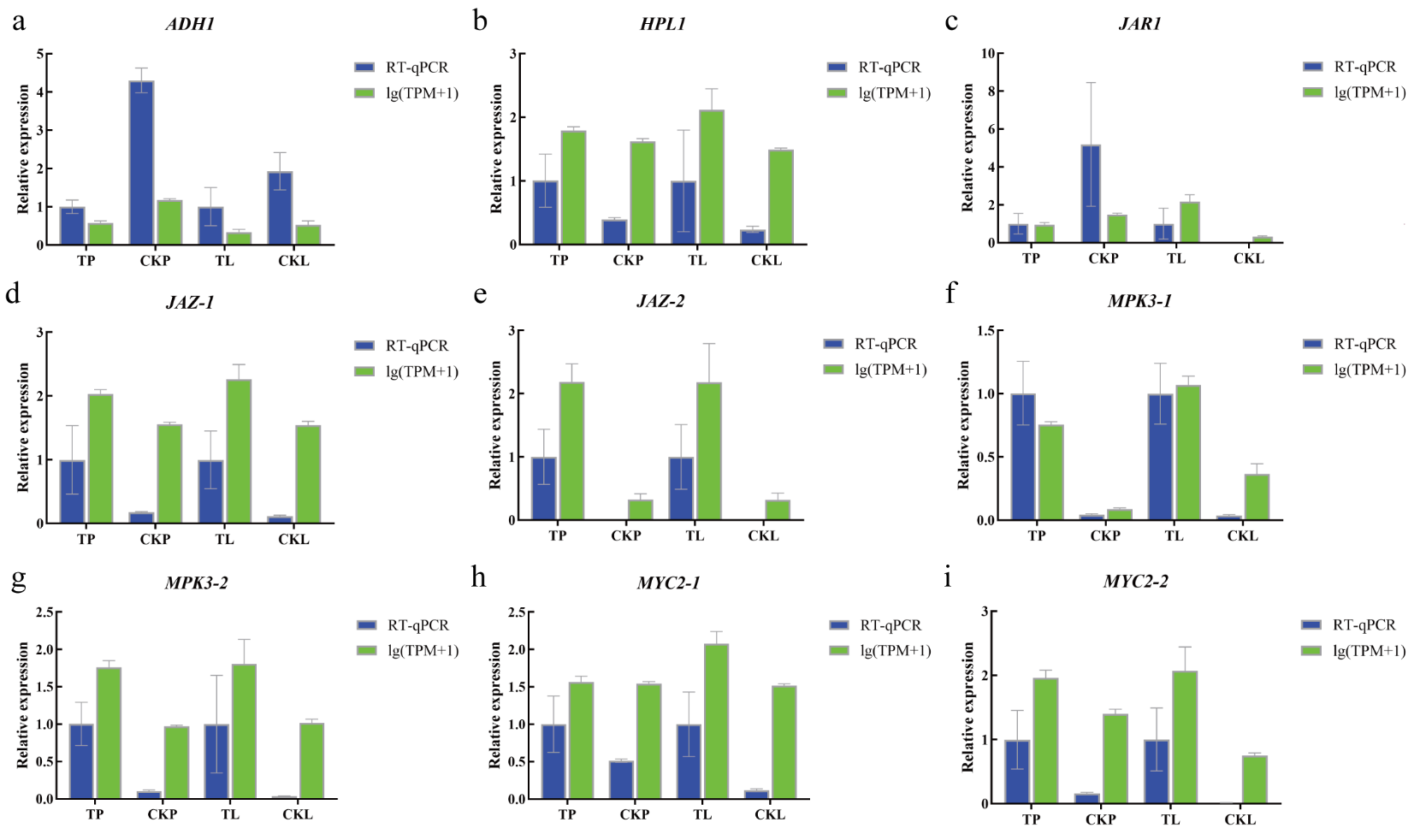


**Supplementary Figure 2.** Quantitative RT-PCR (qRT-PCR) validation of nine differentially expressed genes chosen from the transcriptome.

**
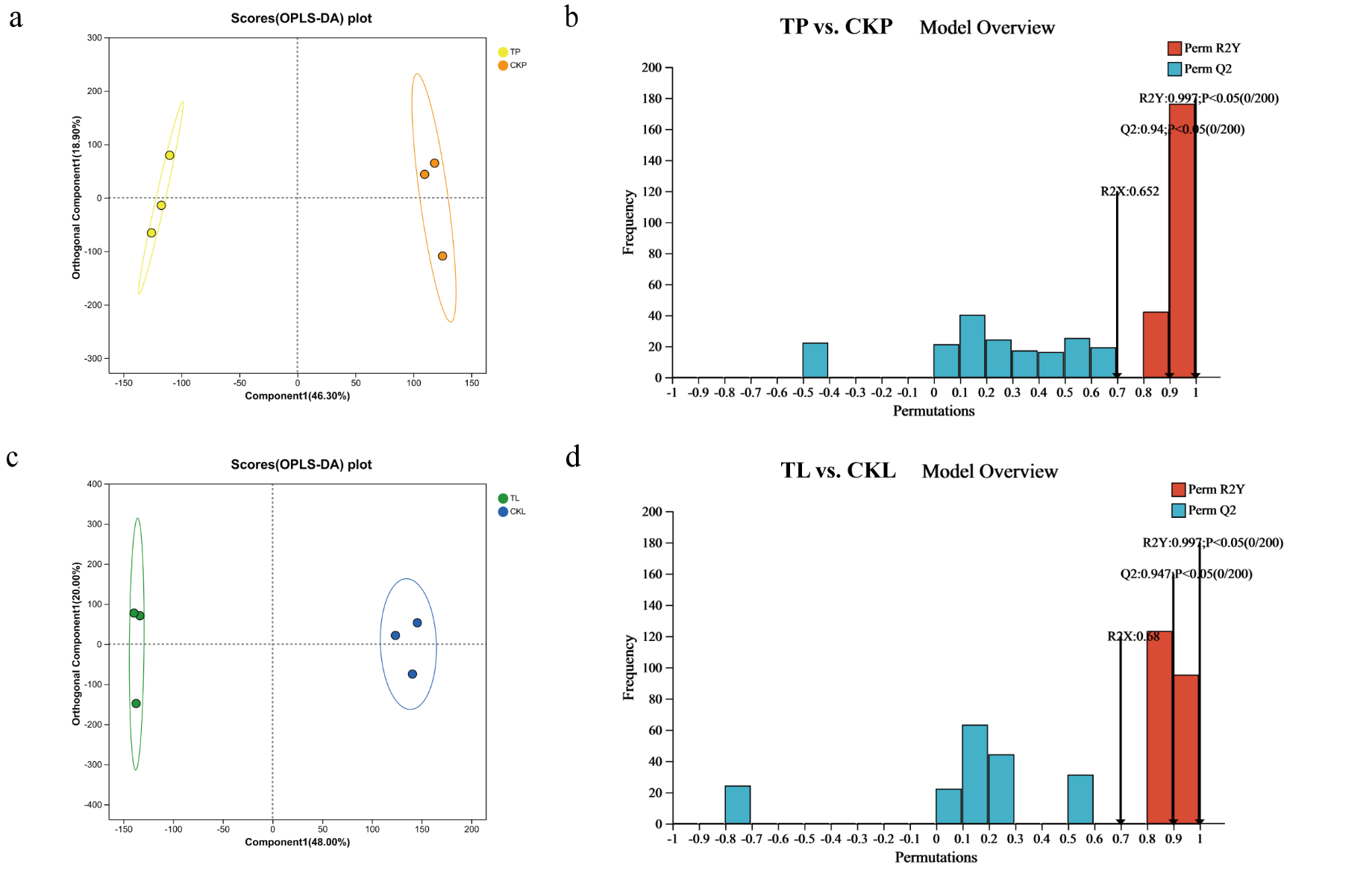
Supplementary Figure 3.** OPLS-DA score and validation plots of the OPLS-DA model by permutation testing (n = 200) for pairwise comparisons among different groups.


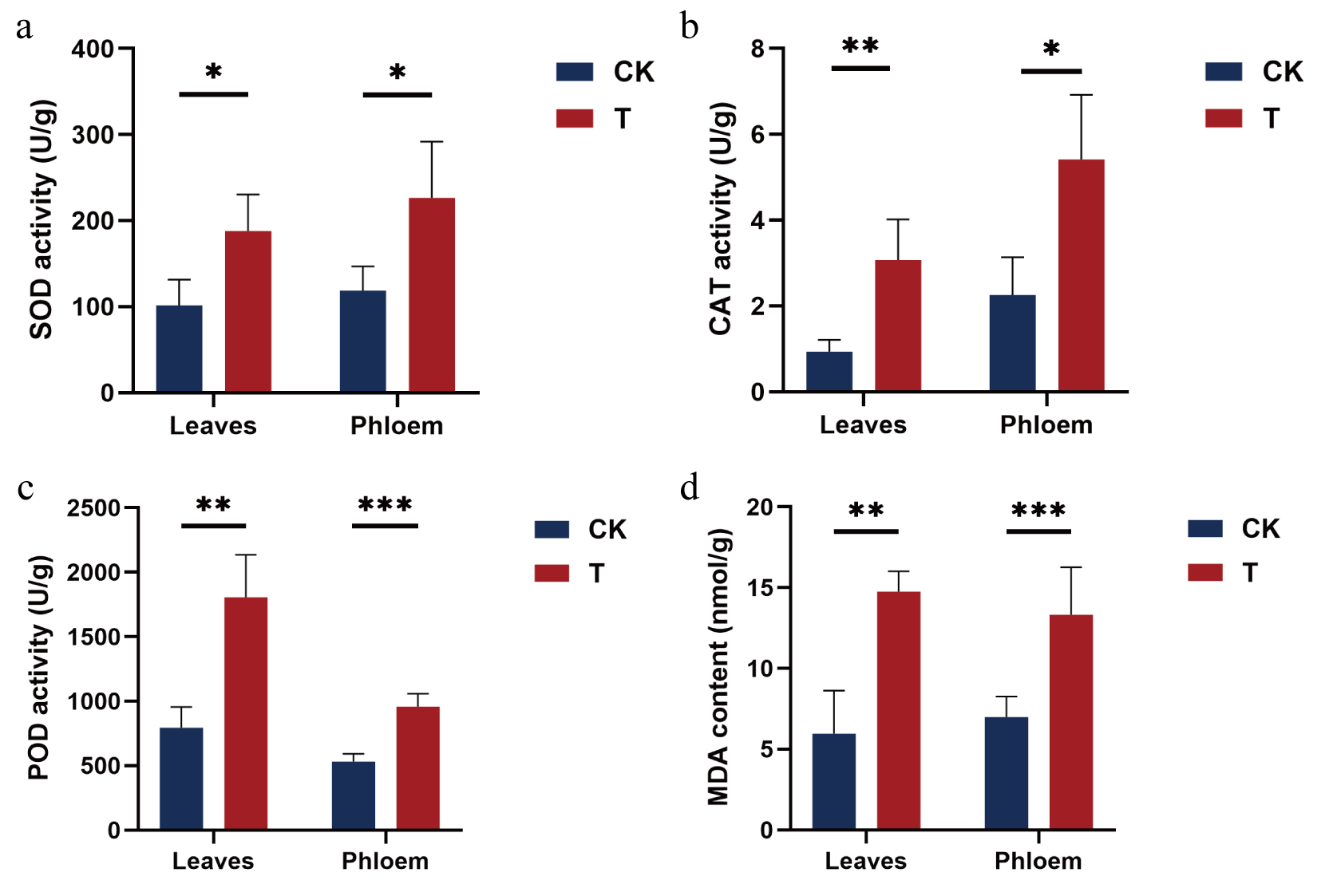
**Supplementary Figure 4. S**OD activity (a), CAT activity (b), POD activity(c), and MDA(d) content of *P. deltoides* ‘Shalinyang’ leaves and phloem before and after ingestion by *A. glabripennis*.


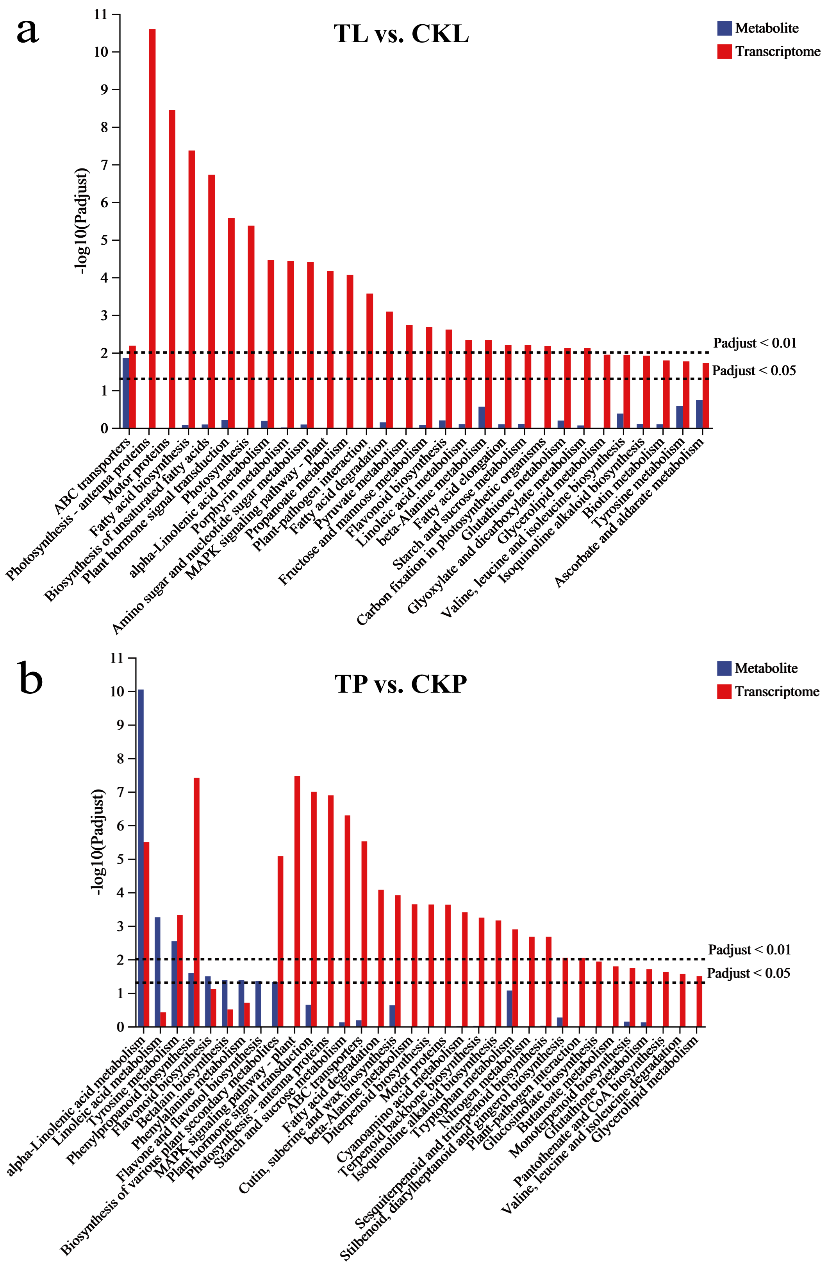


**Supplementary Figure 5.** Co-enrichment pathways of DEGs and DEMs of *P. deltoides* ‘Shalinyang’ leaves(a) and phloem(b) before and after ingestion by *A. glabripennis*.

**
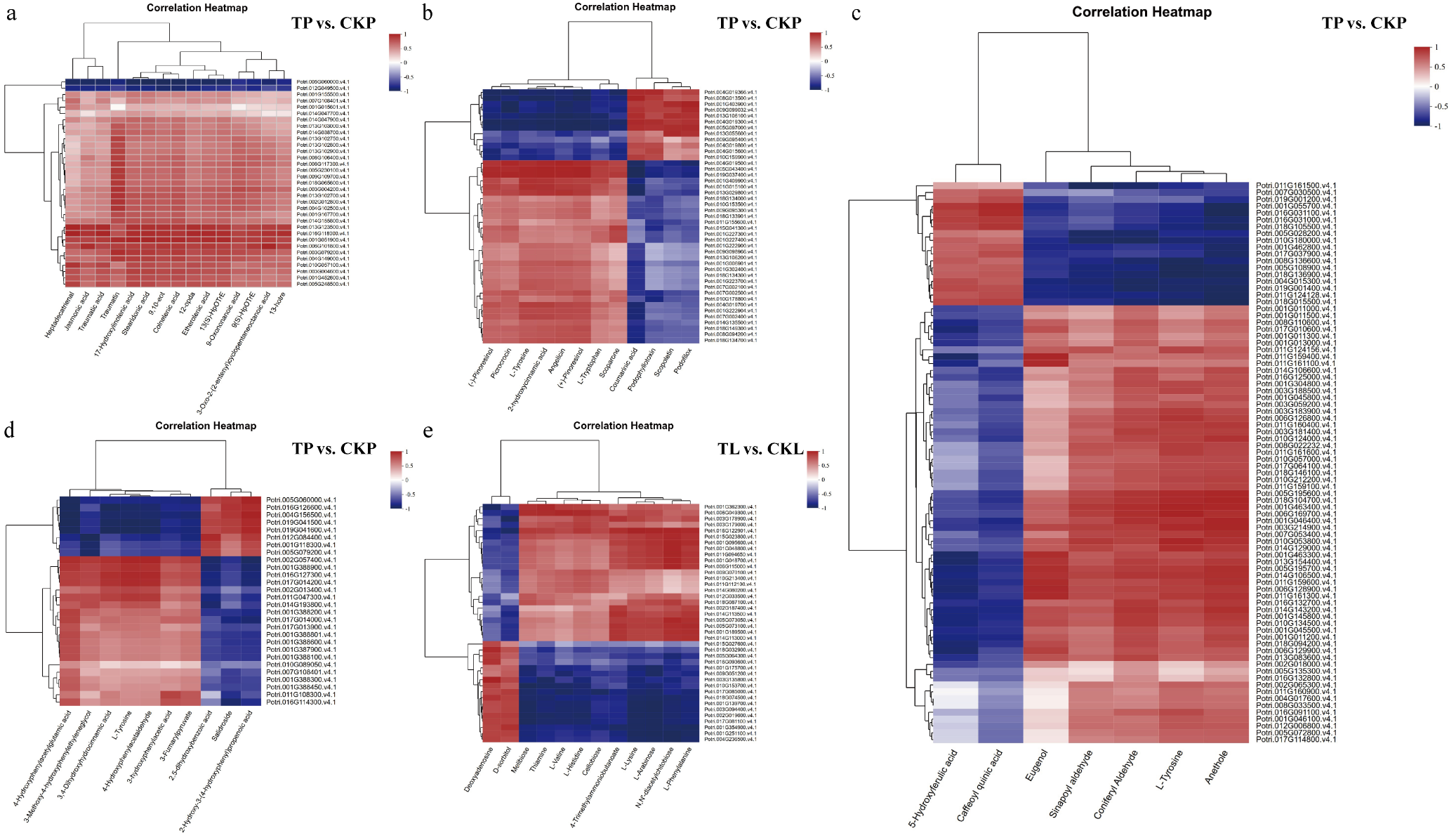
Supplementary Figure 6.** Correlation heatmap between DEGs and DEMs across five significant co-enrichment pathways of *P. deltoides* ‘Shalinyang’ leaves and phloem before and after ingestion by *A. glabripennis*.
